# Maternal hypothyroidism and the risk of preeclampsia: a Danish national and regional study

**DOI:** 10.1186/s40748-024-00186-4

**Published:** 2024-08-02

**Authors:** Maja Hjelm Lundgaard, Marianne Munk Sinding, Anne Nødgaard Sørensen, Aase Handberg, Stig Andersen, Stine Linding Andersen

**Affiliations:** 1 Department of Clinical Biochemistry, Aalborg University Hospital, Hobrovej 18-22, 9000 Aalborg, Denmark; 2 Department of Clinical Medicine, Aalborg University, 9000 Aalborg, Denmark; 3 Department of Obstetrics and Gynecology, Aalborg University Hospital, 9000 Aalborg, Denmark; 4 Department of Geriatrics, Aalborg University Hospital, 9000 Aalborg, Denmark

**Keywords:** Thyroid, Myxedema, Pregnancy, Gestational, Autoimmunity

## Abstract

**Background:**

Maternal hypothyroidism in pregnancy has been proposed to increase the risk of preeclampsia, but uncertainties persist regarding the underlying causal mechanisms. Thus, it remains unclear if an increased risk of preeclampsia in hypothyroid pregnant women is caused by the lack of thyroid hormones or by the autoimmunity per se.

**Methods:**

We conducted a retrospective study of two pregnancy cohorts in the Danish population. The nationwide cohort (*n* = 1,014,775) was register-based and included all singleton pregnancies in Denmark from 1999–2015. The regional cohort (*n* = 14,573) included the biochemical measurement of thyroid stimulating hormone (TSH), thyroid peroxidase antibodies (TPO-Ab), and thyroglobulin antibodies (Tg-Ab) (ADVIA Centaur XPT, Siemens Healthineers) among pregnant women in The North Denmark Region from 2011–2015 who had a blood sample drawn in early pregnancy as part of routine prenatal screening for chromosomal anomalies. The associations between diagnosed and biochemically assessed hypothyroidism and a diagnosis of preeclampsia were evaluated using logistic regression (adjusted odds ratio (aOR) with 95% confidence interval (CI)) adjusting for potential confounders, such as maternal age, diabetes, and parity.

**Results:**

In the nationwide cohort, 2.2% of pregnant women with no history of hypothyroidism (reference group (ref.)) were diagnosed with preeclampsia, whereas the prevalence was 3.0% among pregnant women with hypothyroidism (aOR 1.3 (95% CI: 1.2–1.4)) and 4.2% among women with newly diagnosed hypothyroidism in the pregnancy (aOR 1.6 (95% CI: 1.3–2.0)). In the regional cohort, 2.3% of women with early pregnancy TSH < 2.5 mIU/L (ref.) were diagnosed with preeclampsia. Among women with TSH ≥ 6 mIU/L, the prevalence was 6.2% (aOR 2.4 (95% CI: 1.1–5.3)). Considering thyroid autoimmunity, preeclampsia was diagnosed in 2.2% of women positive for TPO-Ab (> 60 U/mL) or Tg-Ab (> 33 U/mL) in early pregnancy (aOR 0.86 (95% CI: 0.6–1.2)).

**Conclusions:**

In two large cohorts of Danish pregnant women, maternal hypothyroidism was consistently associated with a higher risk of preeclampsia. Biochemical assessment of maternal thyroid function revealed that the severity of hypothyroidism was important. Furthermore, results did not support an association between thyroid autoimmunity per se and preeclampsia.

## Background

Hypothyroidism is a common endocrine disorder and constitutes a substantial burden of chronic disease in pregnant women [1]. Hypothyroidism in women of reproductive age is predominantly caused by autoimmune mechanisms, and thyroid peroxidase autoantibodies (TPO-Ab) as well as thyroglobulin autoantibodies (Tg-Ab) are the key markers of underlying thyroid autoimmunity [2]. Uncertainties prevail regarding the clinical management of subtypes of hypothyroidism in pregnant women [1, 3]. It is well-established that overt hypothyroidism should be treated to prevent maternal and fetal complications, however, the management of smaller aberrations in maternal thyroid function is a matter of debate [1, 4].

One of the concerns regarding undiagnosed and untreated maternal hypothyroidism relates to the risk of pregnancy complications [1]. Thyroid hormones play a significant role for the normal function of the placenta during a pregnancy and lack of thyroid hormones could be a cause of placental dysfunction [5], which may lead to adverse pregnancy outcomes such as preeclampsia and intrauterine growth restriction [6]. Uncertainties persist regarding the underlying causal mechanisms. Thus, it remains unclear if an increased risk of preeclampsia in hypothyroid pregnant women is caused by the lack of thyroid hormones or by the autoimmunity per se.

This study aimed to evaluate the association between maternal hypothyroidism in early pregnancy and outcome of preeclampsia in Danish pregnant women with the use of a nationwide register-based cohort and a regional biobank. More specifically, the aim was to evaluate the role of abnormal maternal thyroid function as opposed to abnormal maternal thyroid autoimmunity and to consider the severity of maternal hypothyroidism in the associations observed.

## Methods

We conducted a retrospective study of two cohorts with linkage to information in nationwide registers. The nationwide cohort (*n* = 1,014,775) included all singleton births in Denmark from 1999 to 2015 who were registered in the Danish Medical Birth Register (MBR) [7]. The regional cohort (*n* = 14,573) included pregnant women in the North Denmark Region from June 2011 to January 2015 who had a blood sample drawn in early pregnancy (median pregnancy week 10) as part of routine prenatal screening for chromosomal anomalies. Thus, the samples were drawn for non-thyroidal reasons, were stored in a biobank, and later used for the measurement of a series of thyroid parameters [8]. In this study, the first blood sample during each pregnancy in the study period was included among pregnancies with an outcome of singleton birth. The study was approved by the North Denmark Region Committee on Health Research Ethics (N-20150015) and registered according to the General Data Protection Regulation in the North Denmark Region (2015–34; 2016–76).

Information on all births in Denmark was obtained from the MBR including maternal age, parity, smoking in pregnancy, pre-pregnancy body mass index (BMI), gestational age at birth, gender of the child, birth year, birth weight as well as paternal age. Information on the diagnoses of disease was assessed from the Danish Nationwide Hospital Register [9] which contains all in- and outpatient visits to Danish hospitals coded according to the 10th International Classification of Disease (ICD-10) from 1994 an onwards. Information on redeemed prescriptions of drugs coded according to the Anatomical Therapeutic Chemical Classification System (ATC) was obtained from the Danish Nationwide Prescription Register [10]. Hypothyroidism was defined as a hospital diagnosis of hypothyroidism (ICD-10: all E03 codes) and/or two redeemed prescriptions of thyroid hormone therapy (ATC: H03AA01, H03AA02) up until five years after birth of the child. Onset of maternal hypothyroidism was defined by the time of the first hospital diagnosis or the first redeemed prescription which ever came first and categorized as before the pregnancy, in the pregnancy, and after the pregnancy.

Preeclampsia was defined as a hospital diagnosis of preeclampsia (ICD-10: all O14 codes) and/or eclampsia (ICD-10: all O15 codes) that was registered from pregnancy week 20 until 30 days after birth of the child in the pregnancy under study. In a sub-analysis, gestational hypertension (ICD-10: all O13 codes, all O16 codes) was evaluated as well as the severity of preeclampsia (severe (ICD-10: O14.1–14.2; all O15 codes) or moderate (ICD-10: O14.0)). If more than one of these diagnoses were registered in the pregnancy, the most severe diagnosis was included according to the following hierarchy: severe preeclampsia, moderate preeclampsia, and gestational hypertension. Diabetes was considered as a co-variate in the analyses and defined as one or more of the following registrations up until five years after birth of the child: A hospital diagnosis of diabetes mellitus (ICD-10: all E10-E14 codes) and/or gestational diabetes (ICD-10: all O24 codes) and/or two redeemed prescriptions of antidiabetics (ATC: A10).

The blood samples in the regional cohort were drawn in median pregnancy week 10 (range 4–20), and serum residues were stored at -80 °C until measurement of thyroid-stimulating hormone (TSH), thyroid peroxidase antibodies (TPO-Ab) and thyroglobulin antibodies (Tg-Ab) on an ADVIA Centaur XPT (Siemens Healthineers, Erlangen, Germany) in 2015–2016 [8]. Method- and pregnancy-specific cut-offs for TPO-Ab and Tg-Ab were previously established within the cohort (TPO-Ab: 60 U/mL and Tg-Ab: 33 U/mL) [11] and used for classification of thyroid autoantibody positive women. The severity of maternal hypothyroidism was assessed from categories of maternal TSH.

The associations between maternal hypothyroidism and outcome of preeclampsia were evaluated using multivariate logistic regression (adjusted odds ratio (aOR) with 95% confidence intervals (95% CI)). The adjusted model included sex of the child, parity, origin, smoking in pregnancy, and diabetes (dichotomous variables) as well as pregnancy week of blood sampling, birth year of the child and maternal (continuous variables). In a sub-analysis, we evaluated the association with paternal hypothyroidism since we hypothesized that any underlying autoimmune or genetic component would be reflected in associations with both maternal and paternal hypothyroidism.

Statistical analyses were performed using STATA version 17.0 (Stata Corp LLC, College Station, Texas, USA).

## Results

Altogether 1,014,775 pregnancies were included in the nationwide cohort and 14,573 in the regional cohort (Table 1). As expected, the two cohorts differed on certain maternal characteristics such as smoking in pregnancy and frequency of diabetes which is compatible with the time trend during the 17 years of inclusion in the nationwide cohort. When a sub-cohort of the nationwide study population was identified to match the years of inclusion in the regional cohort, the differences levelled out (Table 1). However, it was a characteristic that women in the regional cohort were younger, less often of non-Danish origin, and more often obese (Table 1).

**Table 1:**
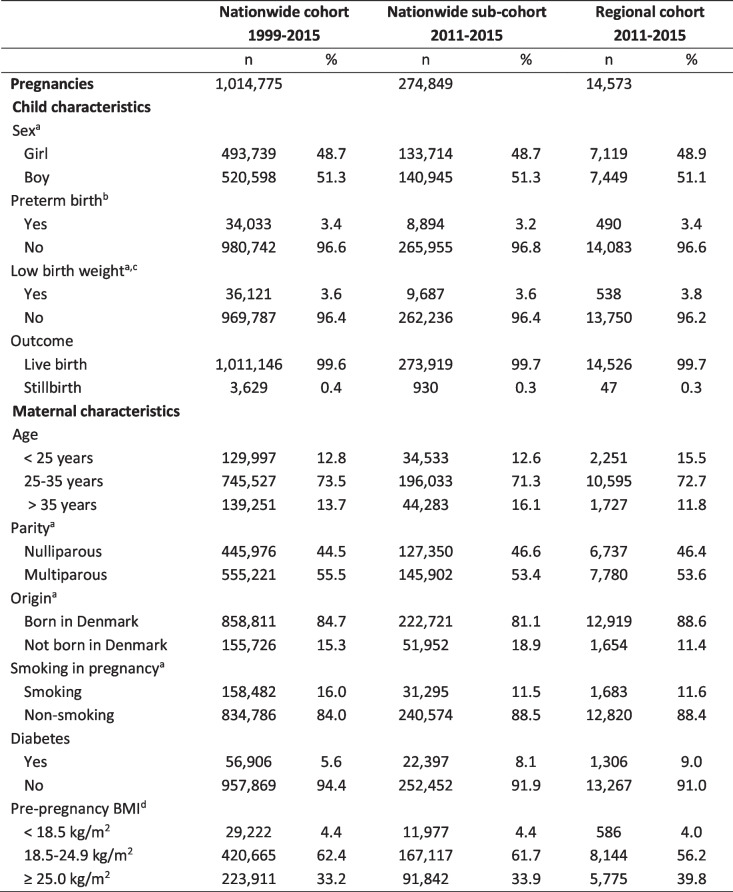
Characteristics of the nationwide cohort and the regional cohort

*Abbreviations: BMI* body mass index

^a^Missing values not included (sex of the child *n* = 438; birth weight *n* = 8,867; parity *n* = 13,578; origin *n* = 238; smoking *n* = 21,507)

^b^Gestational age at birth < 37 full weeks and zero days (37+0). ^c^Birth weight < 2500 grams. ^d^Data only available from 2004 and onwards (Missing values BMI *n* = 340,977)

In the nationwide cohort, 2.2% of pregnant women with no history of hypothyroidism were diagnosed with preeclampsia, whereas the frequency was 3.0% among women with hypothyroidism (Table 2). This overall association revealed a significant higher risk of preeclampsia among women with hypothyroidism in crude and adjusted analyses. Further, a higher risk was observed irrespective of when maternal hypothyroidism was first diagnosed in relation to the pregnancy, however, with a trend towards the highest risk of preeclampsia when maternal hypothyroidism was first time diagnosed in the pregnancy (Table 2). Considering the severity of preeclampsia, maternal hypothyroidism increased the risk of severe as well as moderate preeclampsia (Table 3). However, no significant association with gestational hypertension was seen (Table 3). Most women identified with hypothyroidism had redeemed prescriptions of Levothyroxine (96%) and redeemed more than one prescription in the study period (99%). Overall, results did not change when analyses were restricted to women with redeemed prescriptions or to women with a minimum of two prescriptions.

**Table 2:**
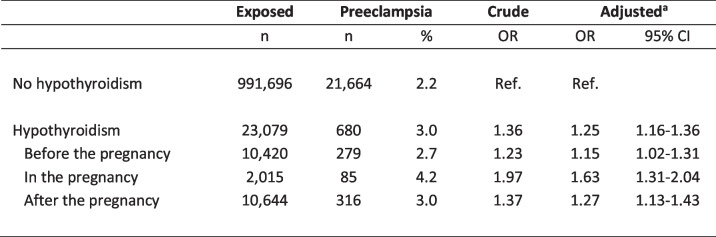
Maternal hypothyroidism and the risk of preeclampsia in the nationwide cohort

*Abbreviations*: *OR* odds ratio, *CI* confidence interval, *ref.* reference group

^a^Adjusted model included: birth year and sex of the child, maternal age, parity, origin, smoking, and diabetes

**Table 3:**
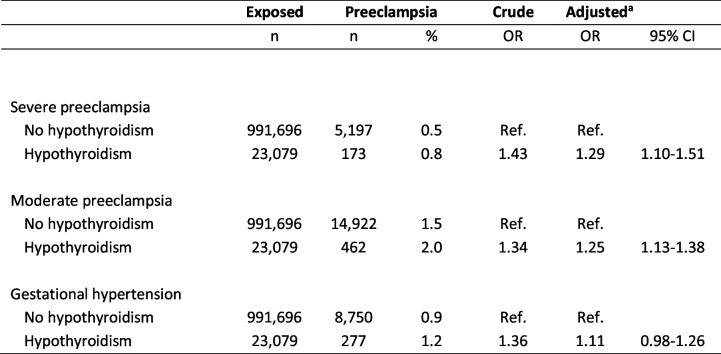
Maternal hypothyroidism and the severity of preeclampsia as well as gestational hypertension in the nationwide cohort

*Abbreviations*: *OR* odds ratio, *CI* confidence interval, *ref.* reference group

^a^Adjusted model included: birth year and sex of the child, maternal age, parity, origin, smoking, and diabetes

**Fig. 1:**
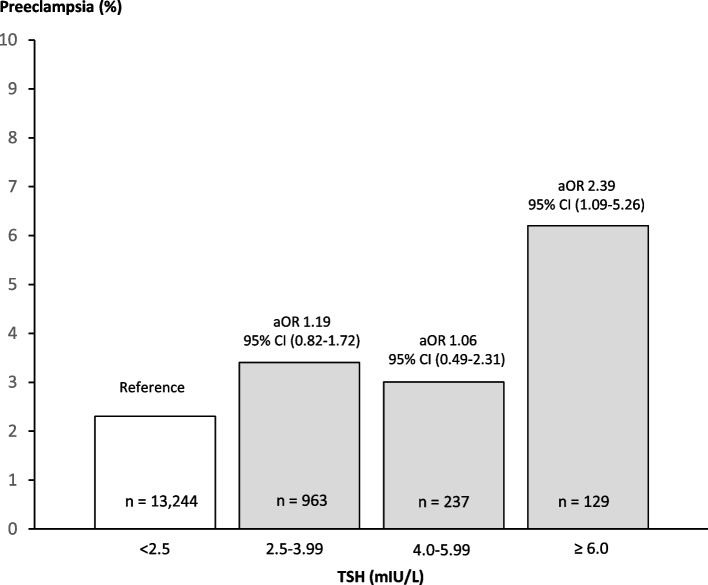
Adjusted odds ratios (aOR) with 95% confidence intervals (95% CI) on the association between maternal thyroid-stimulating hormone (TSH) and the risk of preeclampsia in the regional cohort. The adjusted model included pregnancy week of blood sampling, birth year, sex of the child, maternal age, parity, origin, body mass index, smoking, and diabetes

The findings that maternal hypothyroidism associated with a risk of preeclampsia irrespective of whether maternal thyroid disease was known in pregnancy or later diagnosed led to considerations on the underlying mechanisms. No association with paternal hypothyroidism was seen in the nationwide cohort (Table 4) or with levels of thyroid autoantibodies among women in the regional cohort (Table 5). On the other hand, high levels of maternal TSH (≥ 6 mIU/L) associated with a higher risk of preeclampsia (Fig. 1). Altogether 147 women received treatment with Levothyroxine in the pregnancy, and in 14 women the treatment was started in the pregnancy in median pregnancy week 11 (range 4–24). More specifically, 26 pregnant women (20.2%) with TSH ≥ 6 mIU/L received treatment with Levothyroxine at the time of blood sampling in early pregnancy. When analyses were performed with the additional adjustment for medical treatment in early pregnancy, an increased risk of preeclampsia among women with TSH ≥ 6 mIU/L was found, however, the association did not reach statistical significance (aOR 2.05 (95% CI: 0.92–4.60)).

**Table 4:**
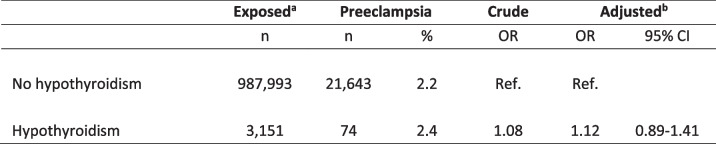
Paternal hypothyroidism and the risk of preeclampsia in the nationwide cohort

*Abbreviations*: *OR* odds ratio, *CI* confidence interval, *ref.* reference group

^a^Pregnancies exposed to maternal hypothyroidism not included in this analysis. Missing information regarding fatherhood *n* = 552

^b^Adjusted model included: birth year and sex of the child, paternal age, origin, and diabetes

**Table 5:**
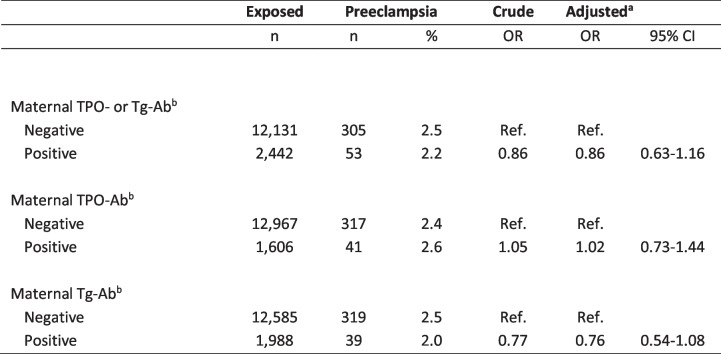
Maternal autoimmunity and the risk of preeclampsia in the regional cohort

*Abbreviations*: *OR* odds ratio, *CI* confidence interval, *ref.* reference group

^a^Adjusted model included: pregnancy week of blood sampling, birth year and sex of the child, maternal age, parity, origin, body mass index, smoking, and diabetes

^b^Cut-off for thyroid autoantibody positivity: TPO-Ab > 60 U/mL and Tg-Ab > 33 U/mL

## Discussion

### Principle findings

In a large nationwide cohort of 1,014,775 pregnancies, women with hypothyroidism more frequently developed preeclampsia, and especially if hypothyroidism was first time diagnosed in the pregnancy. The association was substantiated in a regional cohort of 14,573 pregnancies with biochemical assessment of thyroid parameters in early pregnancy. In this case, elevated maternal TSH (≥ 6 mIU/L) associated with a higher risk of preeclampsia, whereas no association with thyroid autoantibodies was found.

### Interpretation

The risk of adverse pregnancy outcomes in women with hypothyroidism has long been a matter of clinical and scientific focus. From early reports [12], a focus has been on a risk associated with overt and subclinical hypothyroidism. In a recent systematic review and data meta-analysis, it was found that maternal subclinical hypothyroidism was associated with a higher risk of developing preeclampsia [13]. Maternal overt hypothyroidism was not specifically investigated in this study, and women with known thyroid disease were excluded from the analyses, but higher maternal TSH associated with a risk of preeclampsia in continuous analyses [13]. Furthermore, TPO-Ab positivity was not a risk factor for preeclampsia in this large study. Overall, our results add to the existing summarized evidence [13] corroborating an association with maternal hypothyroidism, and not with thyroid autoantibodies per se. Furthermore, results of our study substantiate the level of maternal TSH associated with a higher risk of preeclampsia and turn the focus towards severe maternal hypothyroidism.

Characteristic of the existing literature on maternal hypothyroidism and the risk of preeclampsia is a heterogeneity in the studies regarding exposure and outcome assessment and definition. All studies were observational in design; however, it varies whether exposure and outcome are assessed from diagnosis or biochemical assessment. A strength of our study was the combined use of two pregnancy cohorts and thereby the assessment of the association between maternal hypothyroidism and preeclampsia using two different designs that were giving similar results.

The first cohort that we investigated was a large nationwide and register-based cohort. Nationwide health registers in Denmark provide unique opportunities for identification of all pregnant women and linkage to information on exposure and outcome. In the nationwide cohort, we found that maternal hypothyroidism associated with a higher risk of preeclampsia, and this association has also been found in other large register-based birth cohorts [14–16]. Such register-based designs provide large cohorts and many exposed pregnancies; however, the exposure is indirectly assessed from registrations of hospital diagnoses and redeemed prescriptions of drugs, and we can only speculate on the underlying mechanisms of the associations observed. A pertinent question regarding hypothyroidism in pregnant women is on the differential pathogenic role of abnormal thyroid function and thyroid autoantibodies as the dominant cause of hypothyroidism is autoimmune disease in women of this age span. To elaborate further on these differential components in the register-based design we evaluated results according to the registered onset of maternal hypothyroidism and according to paternal hypothyroidism. If autoimmune mechanisms are dominant factors in the higher risk of preeclampsia association with hypothyroidism, we would expect an association with maternal hypothyroidism irrespective of when the disorder was diagnosed in relation to the pregnancy under study. Furthermore, we would expect an association with paternal hypothyroidism as a marker of an autoimmune and genetic component. We found an association between maternal hypothyroidism and preeclampsia both when maternal hypothyroidism was first time registered before, during and after the pregnancy, however, the association was most pronounced when maternal hypothyroidism was newly diagnosed in the pregnancy. On the other hand, no association between paternal hypothyroidism and preeclampsia was seen. Thus, our findings in the register-based cohort were not completely clear but pointed towards an association with abnormal maternal thyroid function rather than thyroid autoimmunity. This hypothesis would align with a high risk among women newly diagnosed with hypothyroidism in the pregnancy because euthyroidism is not immediately obtained. Furthermore, we previously showed that the frequency of having abnormal thyroid function in the pregnancy is high among women with known hypothyroidism (likely insufficiently treated) and among women first diagnosed after the pregnancy (likely undiagnosed and untreated in the pregnancy) [17].

To extend the register-based findings from our and previous studies, a more direct assessment of maternal thyroid function and thyroid autoimmunity in pregnancy is warranted to substantiate the hypothesis. Thus, we included a second cohort in the present study which was a regional pregnancy cohort with blood samples from early pregnant women stored in a biobank and used for measurement of TSH and thyroid autoantibodies. In the regional cohort, we found that women with TSH ≥ 6 mIU/L in early pregnancy had a higher risk of preeclampsia, and these results corroborated our initial findings from the register-based cohort on the association between maternal hypothyroidism and preeclampsia. A similar association between maternal hypothyroidism and outcome of preeclampsia has been presented in other cohort studies with biochemical assessment of TSH in early pregnancy [18, 19]. The definition of hypothyroidism in pregnant women and hereby the level of TSH associated with adverse outcomes and a need for treatment is a matter of debate [1, 3]. We previously evaluated the consistency in maternal biochemical hypothyroidism with repeated blood samples among women in the regional cohort [20] and found that maternal TSH > 6 mIU/L was associated with consistent hypothyroidism whereas smaller abnormalities in maternal TSH less often persisted with repeated blood sampling [20]. Furthermore, we found for other adverse outcomes of pregnancy [21] and child development [22] that markedly elevated maternal TSH was the main risk factor.

An important clinical perspective is whether the treatment of maternal hypothyroidism in pregnancy would reduce the risk of developing preeclampsia. Via linkage to the nationwide health registers in Denmark, we had the possibility to identify women in the regional biobank who had known and treated biochemical hypothyroidism in the early pregnancy sample and those who had undetected hypothyroidism left untreated in the pregnancy. We found a non-significant higher risk of preeclampsia even if the analyses were adjusted for treatment with Levothyroxine. In contrast, other studies showed that women with hypothyroidism who received Levothyroxine in pregnancy did not have a higher frequency of preeclampsia [16, 19]. The evaluation of treatment is, however, difficult in a retrospective, observational design, and even if we identified women treated, we do not know about the duration of abnormal thyroid function in the pregnancy because the biobank assessment relied on a single sample. Thus, the lack of treatment effect seen in our study may relate to the small sample size or reflect the fact that thyroid function was abnormal in critical parts of the pregnancy also among women treated. The risk of insufficient treatment among women with hypothyroidism who becomes pregnant is a concern [17], and more studies on the timing of treatment and the duration of abnormal thyroid function in relation to adverse outcomes among women with hypothyroidism are warranted to extend the findings.

Besides the severity of hypothyroidism, the role of thyroid autoimmunity in relation to adverse pregnancy outcomes remains controversial [1, 3]. This led us to evaluate thyroid autoantibodies (TPO-Ab and Tg-Ab) among pregnant women in the regional cohort, and we did not observe that the thyroid autoantibodies associated with an increased risk of preeclampsia. This finding is in accordance with previous studies and with our hypothesis and findings in the register-based cohort [18, 19, 23].

The underlying mechanisms for an association between hypothyroidism and preeclampsia are still to be elucidated. One theory considers an imbalance in circulating angiogenic factors which in turn is thought to influence the development of preeclampsia and other pregnancy complications [24–26]. Placental dysfunction has been associated with elevated levels of the antiangiogenic factor soluble fms-like tyrosine kinase-1 (sFlt-1), which antagonize the effect of angiogenic signaling from placental growth factor and vascular endothelial growth factor [24–26]. Sparse evidence is available on the association between levels of thyroid hormones and placental markers, but increasing levels of sFlt-1 in pregnant women with subclinical hypothyroidism has been proposed [27, 28]. More studies are needed to evaluate whether the adverse impact of maternal hypothyroidism in pregnancy could be mediated via alterations in placental biomarkers and thereby in placental function.

### Methodological comments

A large nationwide cohort was studied via the use of Danish nationwide health registers and the validity of these register data is considered high [9, 10]. Levothyroxine is a disease-specific treatment of hypothyroidism which requires a prescription. Thus, the combination of hospital diagnoses and redeemed prescriptions of drugs ensured that hypothyroid women managed and treated in general practice were also included in the study. We cannot exclude misclassification of exposure or outcome in the nationwide cohort, and that this misclassification could be differential if a diagnosis of hypothyroidism increases the clinical awareness of the detection of preeclampsia or vice versa. However, we additionally studied the regional cohort which was a retrospective biobank and thereby thyroid function was assessed after the pregnancy and independent of clinical practice. The severity of maternal hypothyroidism was categorized according to maternal TSH in the regional cohort where a high level of TSH > 6 mIU/L associated with a higher risk of preeclampsia, but numbers in this sub-group were too small to stratify and evaluate associations in further subdivision into perspectives such as severity of preeclampsia, status of thyroid autoimmunity, and treatment with Levothyroxine. The inclusion criterion of the regional cohort was that the pregnant women had a blood sample drawn in early pregnancy as part of prenatal screening for chromosomal anomalies, and in this screening program the rate of participation is high [29]. The blood samples were stored at -80 °C for a maximum of five years until further analysis, and the measured thyroid function parameters and thyroid autoantibodies are known to be stable for long-term frozen storage [30].

## Conclusions

In two large cohorts of Danish pregnant women, maternal hypothyroidism was associated with a higher risk of preeclampsia. Biochemical assessment of maternal thyroid function revealed that the severity of hypothyroidism was important. Furthermore, results did not support an association between thyroid autoimmunity per se and preeclampsia.

## Data Availability

No datasets were generated or analysed during the current study.

## Abbreviations

TSH: Thyroid stimulating hormone
TPO-Ab: Thyroid peroxidase antibodies
Tg-Ab: Thyroglobulin antibodies
MBR: The Danish Medical Birth Register
BMI: Body mass index
ICD-10: The 10th International Classification of Disease
ATC: The Anatomical Therapeutic Chemical Classification System
OR: Odds ratio
aOR: Adjusted odds ratio
CI: Confidence interval
Ref.: Reference group
sFlt-1: Soluble fms-like tyrosine kinase-1
